# Supplementary material for: SENP1 prevents steatohepatitis by suppressing RIPK1-driven apoptosis and inflammation
Source: Nat Commun. 2022 Nov 22;13:7153. doi: 10.1038/s41467-022-34993-0 (PMC9681887; doi:10.1038/s41467-022-34993-0)
Supplement: Supplementary file 1 — Supplementary Information [file 41467_2022_34993_MOESM1_ESM.pdf]

SUPPLEMENTARY INFORMATION

**Title:**

# SENP1 prevents steatohepatitis by suppressing RIPK1-driven apoptosis and inflammation

**Author list:**

Lingjie Yan[1,2,9], Tao Zhang[3,9], Kai Wang[4,5,9], Zezhao Chen[1,2], Yuanxin Yang[1,2], Bing Shan[1], Qi Sun[1], Mengmeng Zhang[1], Yichi Zhang[6], Yedan Zhong[1], Nan Liu[1,7], Jinyang Gu[8,6*], & Daichao Xu[1,7*].

**Affiliations:**

[1]Interdisciplinary Research Center on Biology and Chemistry, Shanghai Institute of Organic Chemistry, Chinese Academy of Sciences, Shanghai, 201210, China.

[2]University of Chinese Academy of Sciences, Beijing, 100049, China.

[3]Department of Pathology, Beth Israel Deaconess Medical Center, Harvard Medical School, Boston, MA 02215, USA.

[4]Department of Hepatobiliary and Pancreatic Surgery, Key Laboratory of Integrated Oncology and Intelligent Medicine of Zhejiang Province, Hangzhou First People's Hospital Affiliated Zhejiang University School of Medicine, Hangzhou, 310006, China.

[5]Institute of Organ Transplantation, Zhejiang University, Hangzhou, 310003, China.

[6]Department of Transplantation, Xinhua Hospital Affiliated to Shanghai Jiao Tong University School of Medicine, Shanghai, 200092, China.

[7]Shanghai Key Laboratory of Aging Studies, Shanghai, 201210, China.

[8]Center for Liver Transplantation, Union Hospital, Tongji Medical College, Huazhong University of Science and Technology, Wuhan, 430022, China.

[9]These authors contributed equally: Lingjie Yan, Tao Zhang, Kai Wang

[*]Corresponding author. Contact email address: gjynyd@126.com, xudaichao@sioc.ac.cn

**Inventory of Supporting Information:**

Supplementary Figures 1 to 9

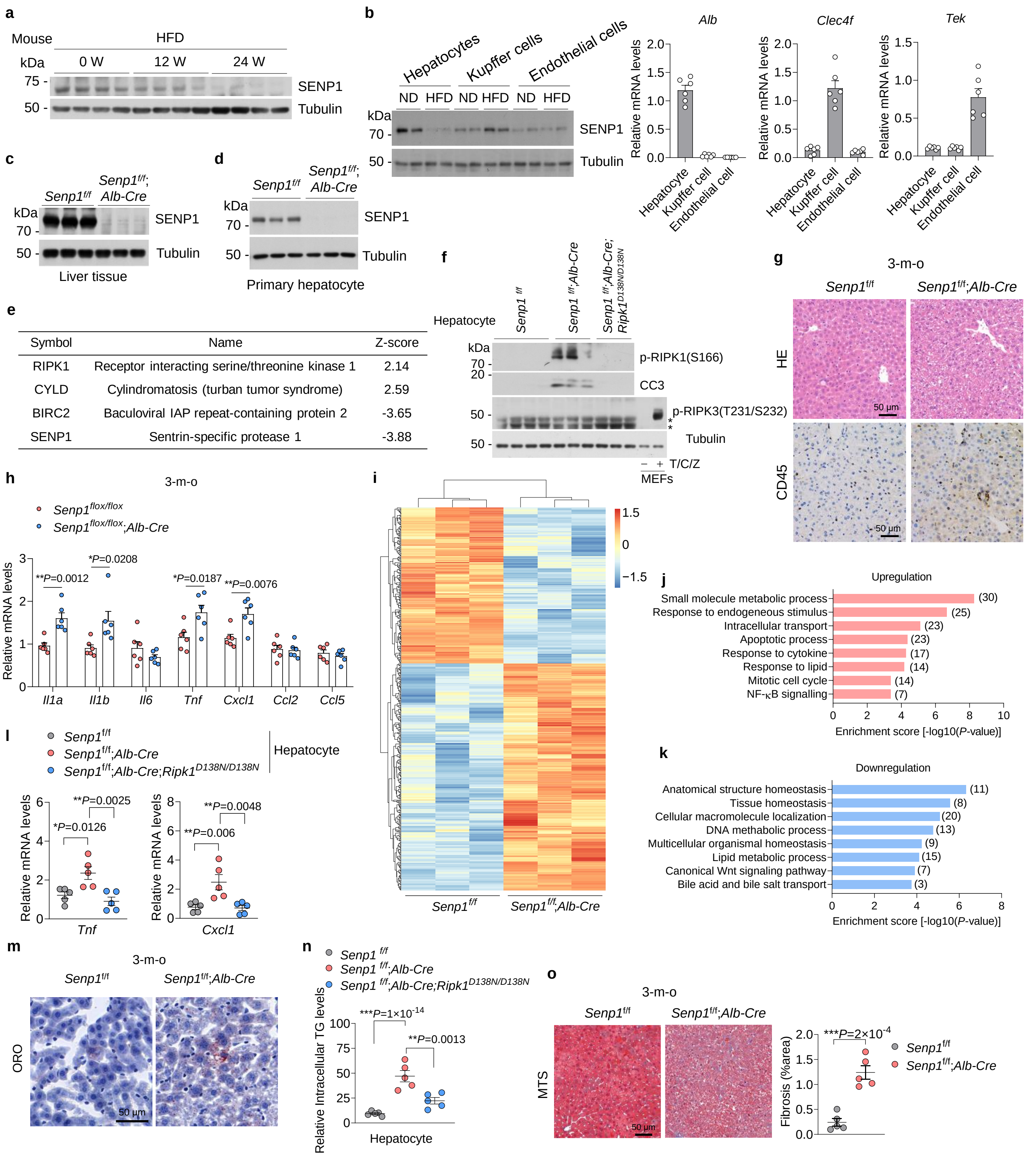

a
Mouse
HFD
0 W
12 W
24 W
kDa
75 -
50 -
SENP1
Tubulin
b
Hepatocytes
Kupffer cells
Endothelial cells
ND HFD ND HFD ND HFD
kDa
70 -
50 -
SENP1
Tubulin
Alb
Clec4f
Tek
Relative mRNA levels
Hepatocyte
Kupffer cell
Endothelial cell
c
Senp1f/f
Senp1f/f; Alb-Cre
SENP1
Tubulin
Liver tissue
d
Primary hepatocyte
e
Symbol | Name | Z-score
RIPK1 | Receptor interacting serine/threonine kinase 1 | 2.14
CYLD | Cylindromatosis (turban tumor syndrome) | 2.59
BIRC2 | Baculoviral IAP repeat-containing protein 2 | -3.65
SENP1 | Sentrin-specific protease 1 | -3.88
f
Hepatocyte
Senp1 f/f
Senp1 f/f;Alb-Cre
Senp1 f/f;Alb-Cre; Ripk1D138N/D138N
p-RIPK1(S166)
CC3
p-RIPK3(T231/S232)
Tubulin
T/C/Z
MEFs
g
3-m-o
Senp1f/f
Senp1f/f;Alb-Cre
HE
CD45
50 μm
h
3-m-o
Senp1flox/flox
Senp1flox/flox;Alb-Cre
Relative mRNA levels
**P=0.0012
*P=0.0208
*P=0.0187
**P=0.0076
Il1a
Il1b
Il6
Tnf
Cxcl1
Ccl2
Ccl5
i
Senp1f/f
Senp1f/f;Alb-Cre
j
Upregulation
Small molecule metabolic process (30)
Response to endogeneous stimulus (25)
Intracellular transport (23)
Apoptotic process (23)
Response to cytokine (17)
Response to lipid (14)
Mitotic cell cycle (14)
NF-κB signalling (7)
Enrichment score [-log10(P-value)]
k
Downregulation
Anatomical structure homeostasis (11)
Tissue homeostasis (8)
Cellular macromolecule localization (20)
DNA methabolic process (13)
Multicellular organismal homeostasis (9)
Lipid metabolic process (15)
Canonical Wnt signaling pathway (7)
Bile acid and bile salt transport (3)
Enrichment score [-log10(P-value)]
l
Senp1f/f
Senp1f/f;Alb-Cre
Senp1f/f;Alb-Cre;Ripk1D138N/D138N
Hepatocyte
Relative mRNA levels
**P=0.0025
*P=0.0126
**P=0.0048
**P=0.006
Tnf
Cxcl1
m
3-m-o
Senp1f/f
Senp1f/f;Alb-Cre
ORO
50 μm
n
Senp1 f/f
Senp1 f/f;Alb-Cre
Senp1 f/f;Alb-Cre;Ripk1D138N/D138N
Relative Intracellular TG levels
***P=1×10-14
**P=0.0013
Hepatocyte
o
3-m-o
Senp1f/f
Senp1f/f;Alb-Cre
MTS
50 μm
Fibrosis (%area)
***P=2×10-4

**Supplementary Figure 1. Deletion of SENP1 in hepatocytes causes RIPK1-driven inflammation and liver damage.**

**a**-**d**, Immunoblotting analysis of SENP1 protein levels in the livers of mice fed a HFD for indicated number of weeks (W) (*n* = 4) (**a**), and isolated hepatocytes, Kupffer cells and endothelial cells from livers of control mice and HFD-fed (24 weeks) mice (*n* = 2) (**b**). The purity of isolated each type of cells was determined by measuring the hepatocyte-specific gene *Alb*, Kupffer cell-specific gene *Clec4f*, and endothelia cell-specific gene *Tek* (*n* = 6) (**b**). **c**, **d**, Immunoblotting analysis of SENP1 expression in livers (**c**) and hepatocytes isolated from livers (**d**) of mice with indicated genotypes (*n* = 3). **e**, The z scores of RIPK1, CYLD, BIRC2 and SENP1 in the genome-wide siRNA screen for regulators of RIPK1-dependent cell death. CYLD and BIRC2 are positive controls. **f**, Immunoblotting analysis of RIPK1 activation, apoptosis and necroptosis in hepatocytes isolated from livers of 8-m-o mice (*n* = 4 for *Senp1*$^{f/f}$, *n* = 3 for *Senp1*$^{f/f}$;*Alb-Cre* and *Senp1*$^{f/f}$;*Alb-Cre*;*Ripk1*$^{D138N/D138N}$). Lysate from MEFs treated with T(10 ng/ml)/CHX(C, 2 μg/ml)/zVAD(Z, 10 μM) for 4 hrs were used as a positive control for p-RIPK3(T231/S232) signal. *: non-specific band. **g**, **h**, Representative images of histological analysis and IHC of CD45 of liver sections (**g**), and quantitative RT-PCR analysis of the mRNA expression of cytokines and chemokines in livers (**h**) from 3-m-o mice (*n* = 6). **i**-**k**, Heatmap (**i**) and GO analysis (**j**, **k**) of genes differentially expressed in livers from 3-m-o mice (*n* = 3). **l**, Quantitative RT-PCR analysis of the mRNA expression of *Tnf* and *Cxcl1* from hepatocytes isolated from 8-m-o mice (*n* = 5). **m**, Oil Red O staining of lipids in livers from 3-m-o mice (*n* = 6). **n**, Relative intracellular TG content in hepatocytes isolated from 8-m-o mice (*n* = 5). **o**, Representative images of Masson's trichrome stained liver sections from 3-m-o mice. The quantification is shown at right (*n* = 5). Data are presented as mean ± s.e.m. (**b**, **h**, **l**, **n**, **o**). One-way ANOVA, post hoc Dunnett's test (**l**, **n**). Unpaired two-tailed t-test (**h**, **o**). Source data are provided in Source Data file.

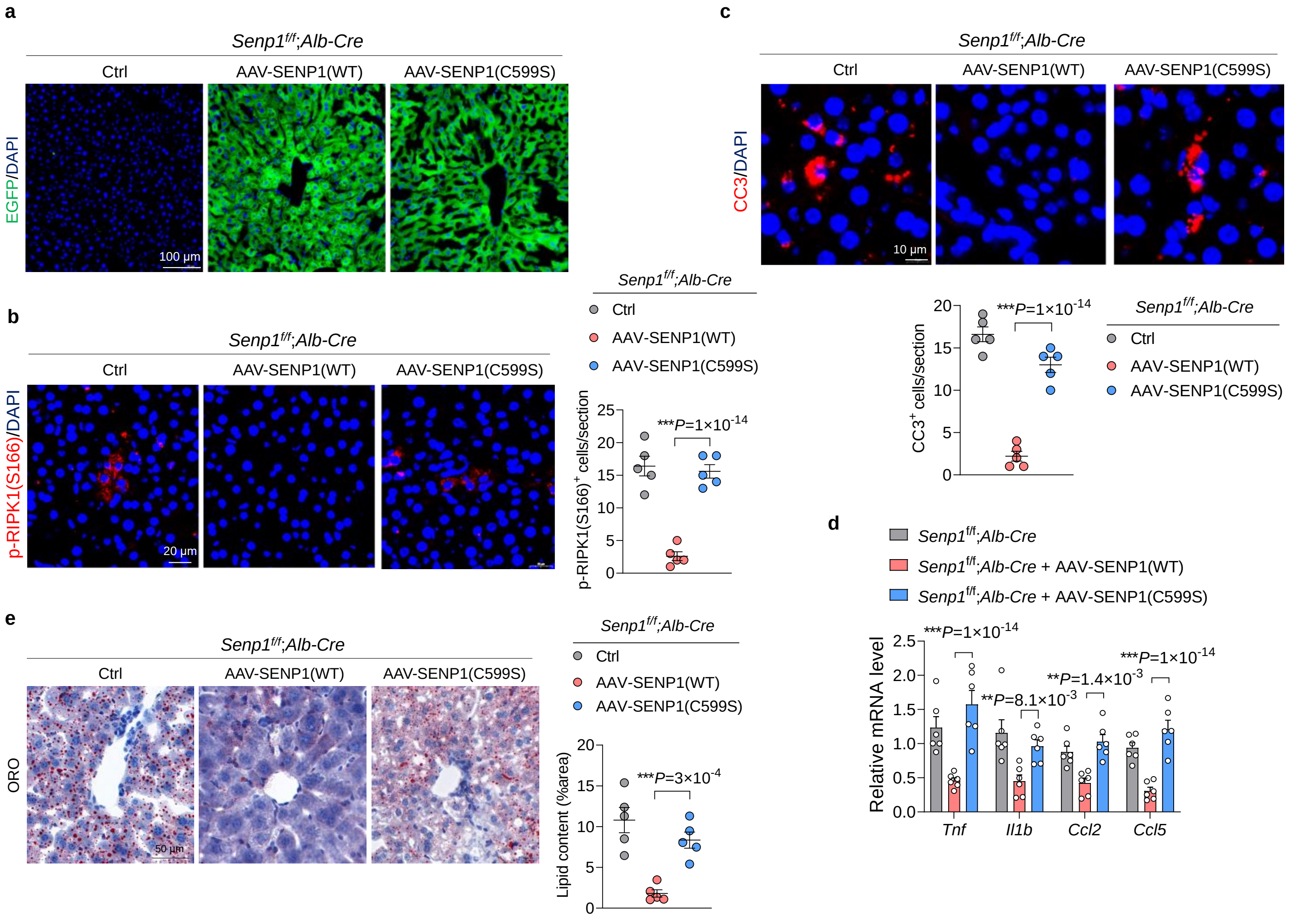
a
Senp1f/f;Alb-Cre
Ctrl
AAV-SENP1(WT)
AAV-SENP1(C599S)
EGFP/DAPI
100 μm
b
Senp1f/f;Alb-Cre
Ctrl
AAV-SENP1(WT)
AAV-SENP1(C599S)
p-RIPK1(S166)/DAPI
20 μm
Senp1f/f;Alb-Cre
Ctrl
AAV-SENP1(WT)
AAV-SENP1(C599S)
p-RIPK1(S166)+ cells/section
***P=1×10-14
c
Senp1f/f;Alb-Cre
Ctrl
AAV-SENP1(WT)
AAV-SENP1(C599S)
CC3/DAPI
10 μm
CC3+ cells/section
***P=1×10-14
Senp1f/f;Alb-Cre
Ctrl
AAV-SENP1(WT)
AAV-SENP1(C599S)
d
Senp1f/f;Alb-Cre
Senp1f/f;Alb-Cre + AAV-SENP1(WT)
Senp1f/f;Alb-Cre + AAV-SENP1(C599S)
Relative mRNA level
***P=1×10-14
**P=8.1×10-3
**P=1.4×10-3
***P=1×10-14
Tnf
Il1b
Ccl2
Ccl5
e
Senp1f/f;Alb-Cre
Ctrl
AAV-SENP1(WT)
AAV-SENP1(C599S)
ORO
50 μm
Senp1f/f;Alb-Cre
Ctrl
AAV-SENP1(WT)
AAV-SENP1(C599S)
Lipid content (%area)
***P=3×10-4

**Supplementary Figure 2. The deSUMOylation activity of SENP1 is required for suppressing RIPK1 activation and liver damage.**

**a**, Representative fluorescent microscopy images out of *n* = 3 livers two months after injections with pAAV-CBh-EGFP-P2A-SENP1-3×FLAG-tWPA or AAV-CBh-EGFP-P2A-SENP1(C599S)-3×FLAG-tWPA. The construct expresses EGFP, which allows for verification of the site and efficacy of infection. **b**, **c**, p-S166 RIPK1 (**b**) and CC3 (**c**) staining of liver sections from *Senp1*$^{f/f}$;*Alb-Cre* control mice, *Senp1*$^{f/f}$;*Alb-Cre*;AAV-*Senp1*$^{WT}$ (SENP1-WT) and *Senp1*$^{f/f}$;*Alb-Cre*;AAV-*Senp1*$^{C599S}$ (SENP1-C599S) mice. Graph depicting numbers of p-S166 RIPK1$^{+}$ staining (**b**) and numbers of CC3$^{+}$ cells (**c**) on liver sections of indicated genotypes, respectively. Each dot represents an individual mouse. Mean ± s.e.m. of *n* = 5 mice for each genotype. One-way ANOVA, post hoc Dunnett's test. **d,** Quantitative RT-PCR analysis of the mRNA expression of cytokines and chemokines in livers of mice with indicated genotypes (*n* = 5 mice for each genotype). Each dot represents an individual mouse. Mean ± s.e.m. One-way ANOVA, post hoc Dunnett's test. **e,** Oil Red O staining of lipids in liver sections from mice of indicated genotypes. Representative images out of *n* = 5 mice for each genotype are represented. Graph depicting percentage of lipid content on liver sections of indicated genotypes. Each dot represents an individual mouse. Mean ± s.e.m. One-way ANOVA, post hoc Dunnett's test. Source data are provided in Source Data file.

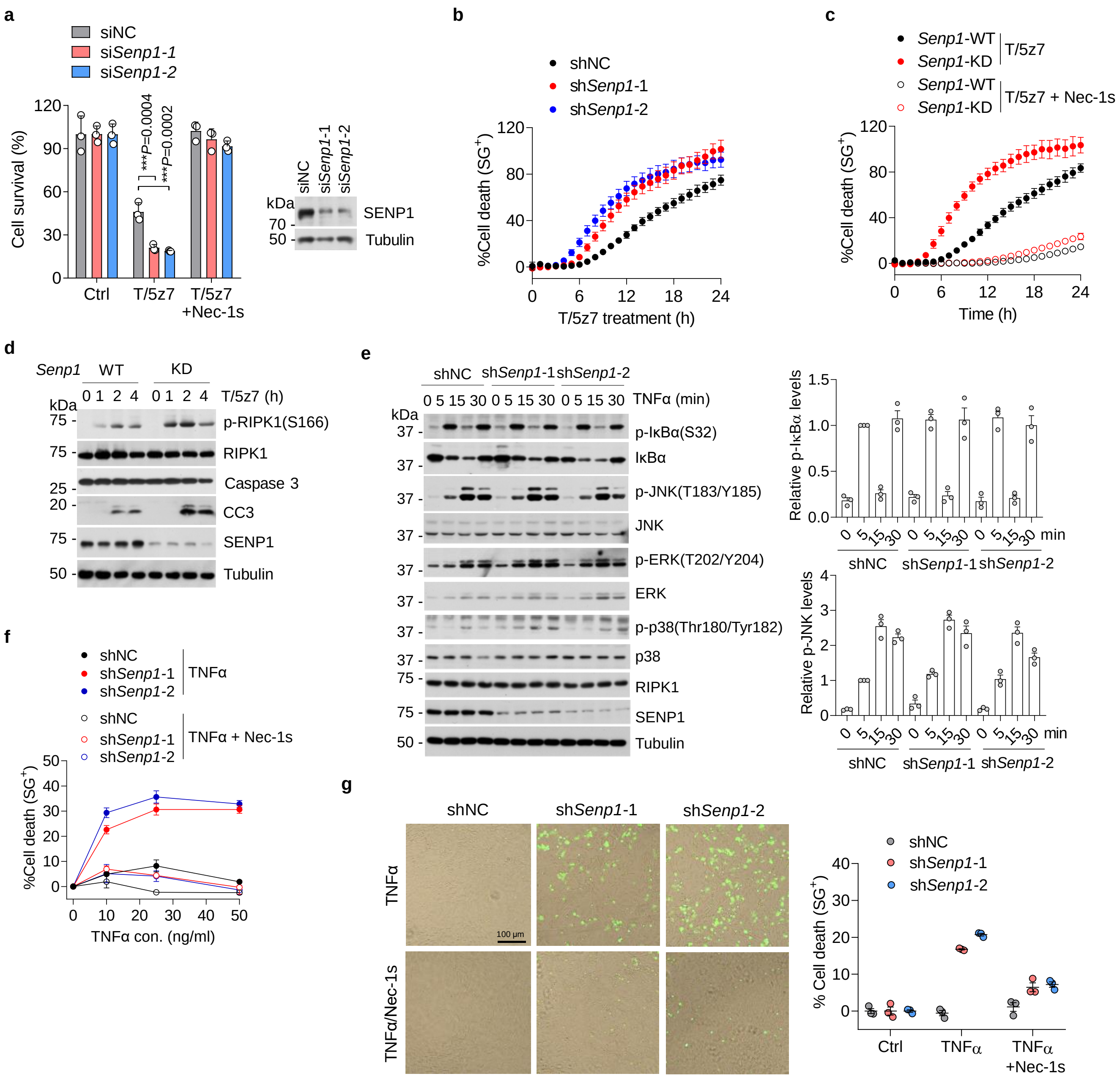
a
siNC
siSenp1-1
siSenp1-2
Cell survival (%)
120
90
60
30
0
***P=0.0004
***P=0.0002
Ctrl
T/5z7
T/5z7
+Nec-1s
siNC
siSenp1-1
siSenp1-2
kDa
70 -
50 -
SENP1
Tubulin
b
shNC
shSenp1-1
shSenp1-2
%Cell death (SG+)
120
80
40
0
0
6
12
18
24
T/5z7 treatment (h)
c
Senp1-WT
Senp1-KD
T/5z7
Senp1-WT
Senp1-KD
T/5z7 + Nec-1s
%Cell death (SG+)
120
80
40
0
0
6
12
18
24
Time (h)
d
Senp1
WT
KD
0 1 2 4 0 1 2 4
T/5z7 (h)
kDa
75 -
p-RIPK1(S166)
75 -
RIPK1
25 -
Caspase 3
20 -
CC3
75 -
SENP1
50 -
Tubulin
e
shNC
shSenp1-1
shSenp1-2
0 5 15 30 0 5 15 30 0 5 15 30
TNFα (min)
kDa
37 -
p-IκBα(S32)
37 -
IκBα
37 -
p-JNK(T183/Y185)
37 -
JNK
37 -
p-ERK(T202/Y204)
37 -
ERK
37 -
p-p38(Thr180/Tyr182)
37 -
p38
75 -
RIPK1
75 -
SENP1
50 -
Tubulin
Relative p-IκBα levels
1.5
1.0
0.5
0.0
0 5 15 30 0 5 15 30 0 5 15 30 min
shNC
shSenp1-1
shSenp1-2
Relative p-JNK levels
4
3
2
1
0
0 5 15 30 0 5 15 30 0 5 15 30 min
shNC
shSenp1-1
shSenp1-2
f
shNC
shSenp1-1
shSenp1-2
TNFα
shNC
shSenp1-1
shSenp1-2
TNFα + Nec-1s
%Cell death (SG+)
50
40
30
20
10
0
0
10
20
30
40
50
TNFα con. (ng/ml)
g
shNC
shSenp1-1
shSenp1-2
TNFα
TNFα/Nec-1s
100 µm
shNC
shSenp1-1
shSenp1-2
% Cell death (SG+)
40
30
20
10
0
Ctrl
TNFα
TNFα
+Nec-1s

**Supplementary Figure 3. Knockdown of SENP1 promotes RIPK1-dependent apoptosis in response to TNFα.**

**a**, MEFs were transfected with siRNAs targeting SENP1, or control (NC), for 48 hrs. The cells were then pre-treated with 5z7 (100 nM) in the presence or absence of Nec-1s (10 μM) for 0.5 hr followed by TNFα (1 ng/ml) for 12 hrs. Cell survival was measured by CellTiter-Glo assay. One-way ANOVA, post hoc Dunnett's test. Knockdown efficiency is shown by immunoblotting with SENP1. **b**, MEFs stably expressing the indicated shRNA were pre-treated with 100 nM 5z7 for 0.5 hr followed by 1 ng/ml TNFα for indicated time. Cell death was measured as a function of time by SytoxGreen positivity assay. **c**, **d**, shNC (hereafter referred to *Senp1*-WT) and sh*Senp1*-2 (hereafter referred to *Senp1*-KD) MEFs were treated with 100 nM 5z7 in the presence or absence of Nec-1s (10 μM) for 0.5 hr followed by 1 ng/ml TNFα for indicated time. Cell death was measured as a function of time by SytoxGreen positivity assay (**c**). The levels of p-S166 RIPK1 and cleaved caspase-3 (CC3) were determined by immunoblotting (**d**). **e**, MEFs stably expressing the indicated shRNA were stimulated with 10 ng/ml TNFα for indicated periods of time and the whole-cell lysates were immunoblotted as indicated. Quantification of relative p-IκBα and p-JNK levels that normalized to Tubulin levels was shown on the right. Mean ± s.e.m. of $n$ = 3 biologically independent experiments. **f**, MEFs stably expressing the indicated shRNA were treated with different concentrations of TNFα for 24 hrs. Cell death was measured by SytoxGreen positivity assay. **g**, MEFs stably expressing the indicated shRNA were treated with TNFα (10 ng/ml) in the presence or absence of Nec-1s (10 μM) for 24 hrs. Representative images of SytoxGreen staining out of three independent experiments are represented. Graph depicting percentage of dead cells. Cell counting was performed manually using ImageJ. Data are presented as mean ± s.d. of three (**a**, **f**, **g**) and six (**b**, **c**) independent samples of one representative experiment out of $n$ = 3 independent experiments. Source data are provided in Source Data file.

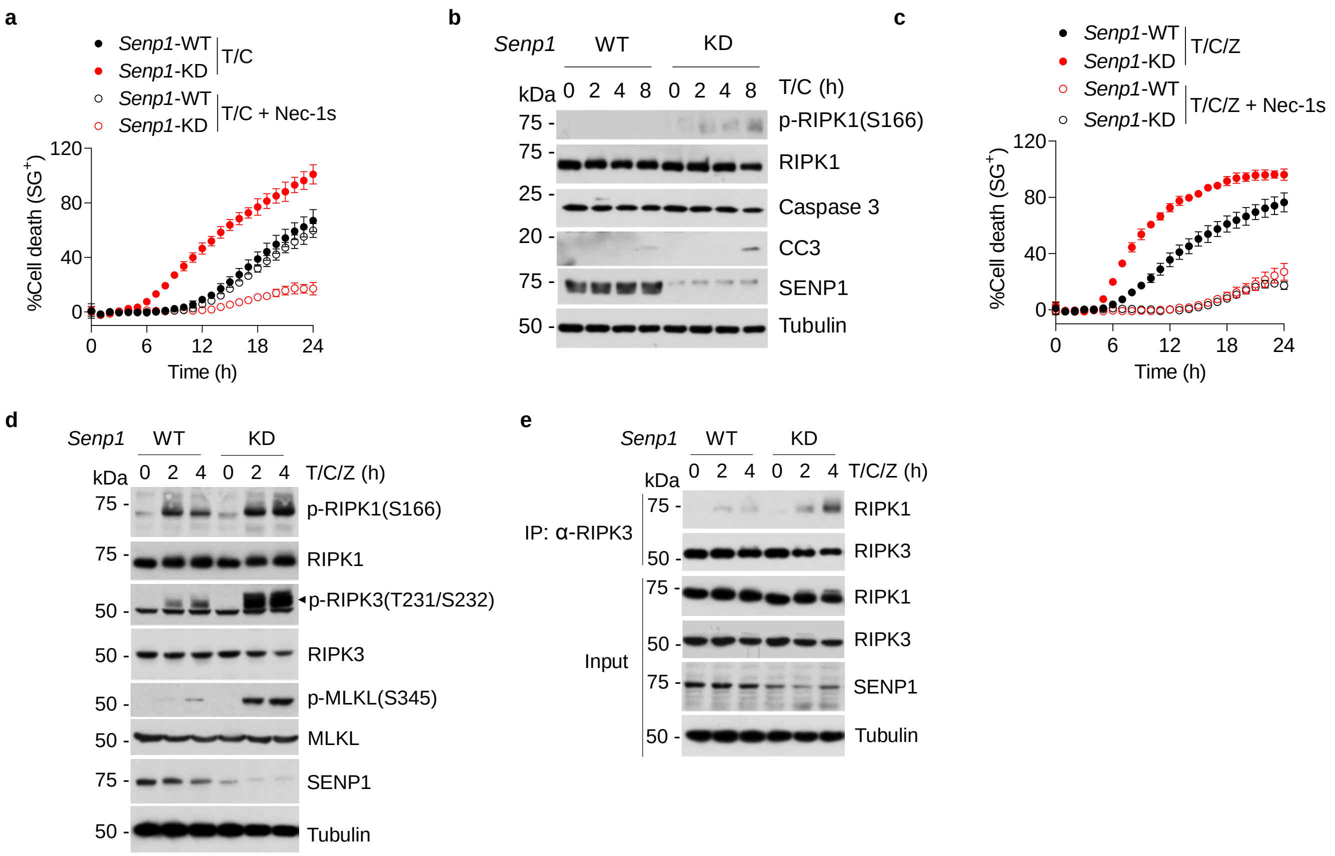
a
Senp1-WT
Senp1-KD
T/C
Senp1-WT
Senp1-KD
T/C + Nec-1s
%Cell death (SG+)
Time (h)
b
Senp1
WT
KD
kDa
T/C (h)
p-RIPK1(S166)
RIPK1
Caspase 3
CC3
SENP1
Tubulin
c
T/C/Z
T/C/Z + Nec-1s
d
T/C/Z (h)
p-RIPK3(T231/S232)
RIPK3
p-MLKL(S345)
MLKL
e
IP: α-RIPK3
Input

**Supplementary Figure 4. Knockdown of SENP1 promotes RIPK1-dependent apoptosis and necroptosis.**

**a**, **b**, SENP1 knockdown sensitized MEFs to T/C-induced cell death and converts RIA to RDA. *Senp1*-WT and *Senp1*-KD MEFs were treated with 2 μg/ml cycloheximide (CHX, C) in the presence or absence of Nec-1s (10 μM) for 0.5 hr followed by 10 ng/ml TNFα for indicated time. Cell death was measured as a function of time by SytoxGreen positivity assay, data are represented as mean ± s.d. of six independent samples of one representative experiment out of *n* = 3 independent experiments (**a**). The levels of p-S166 RIPK1 and CC3 were determined by immunoblotting, similar results were obtained from *n* = 3 independent experiments (**b**). **c**, **d**, SENP1 knockdown sensitized MEFs to T/C/Z-induced necroptosis. *Senp1*-WT and *Senp1*-KD MEFs were treated with CHX (2 μg/ml) and zVAD.fmk (Z, 10 μM) in the presence or absence of Nec-1s (10 μM) for 0.5 hr followed by 1 ng/ml TNFα for indicated time. Cell death was measured as a function of time by SytoxGreen positivity assay, data are represented as mean ± s.d. of six independent samples of one representative experiment out of *n* = 3 independent experiments (**c**). The levels of p-S166 RIPK1, p-T231/S232 RIPK3 and p-S345 MLKL were determined by immunoblotting, similar results were obtained from *n* = 3 independent experiments (**d**). **e**, *Senp1*-WT and *Senp1*-KD MEFs were pre-treated with CHX (C, 2 μg/ml) and zVAD.fmk (Z, 10 μM) for 0.5 hr followed by 1 ng/ml TNFα for indicated time. The necrosome was isolated by immunoprecipitation of RIPK3, and RIPK1 binding was revealed by immunoblotting. Similar results were obtained from *n* = 3 independent experiments. Source data are provided in Source Data file.

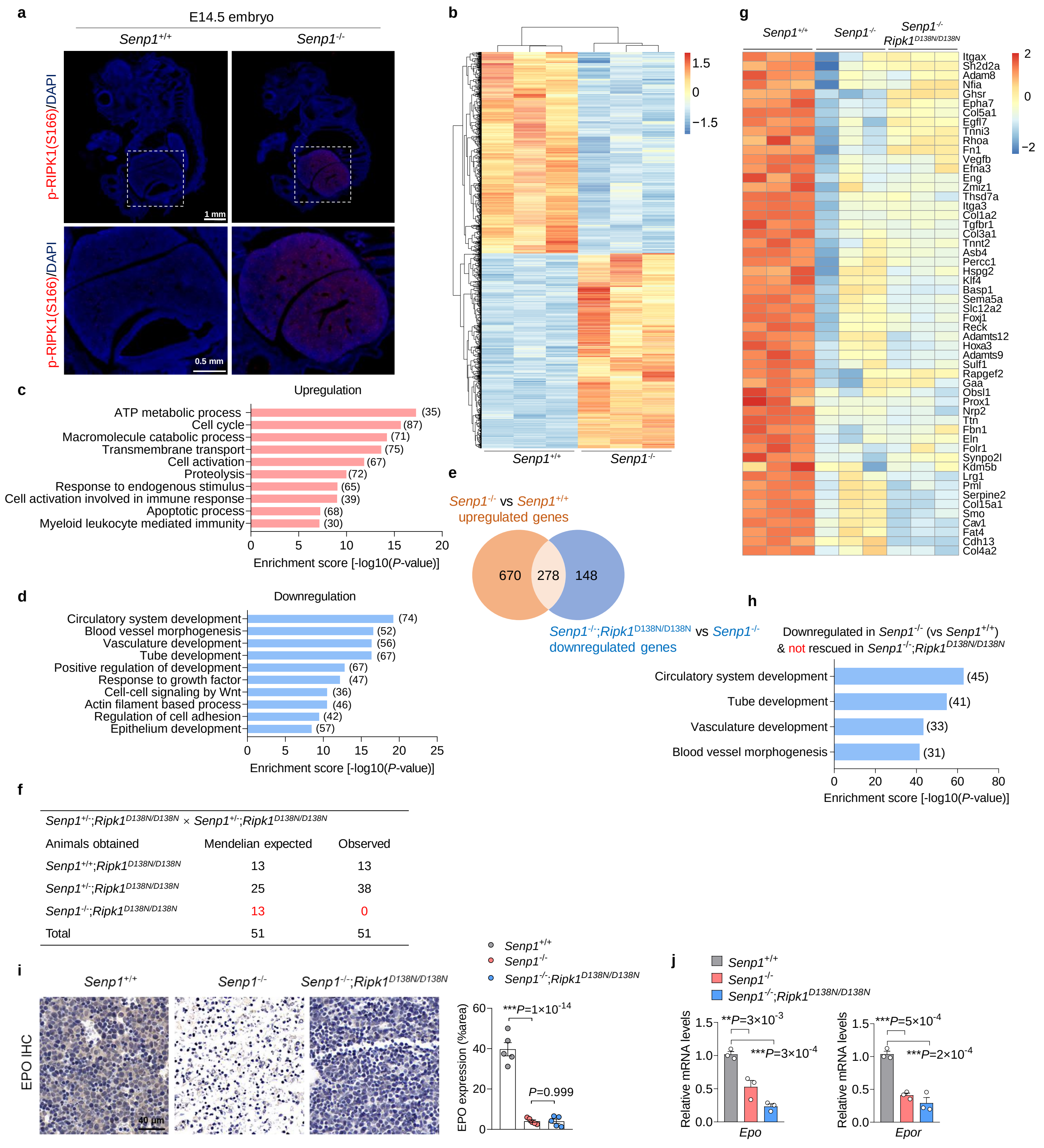
a
E14.5 embryo
Senp1+/+
Senp1-/-
p-RIPK1(S166)/DAPI
1 mm
0.5 mm
b
1.5
0
−1.5
Senp1+/+
Senp1-/-
g
Senp1+/+
Senp1-/-
Senp1-/-
Ripk1D138N/D138N
2
0
−2
Itgax
Sh2d2a
Adam8
Nfia
Ghsr
Epha7
Col5a1
Egfl7
Tnni3
Rhoa
Fn1
Vegfb
Efna3
Eng
Zmiz1
Thsd7a
Itga3
Col1a2
Tgfbr1
Col3a1
Tnnt2
Asb4
Percc1
Hspg2
Klf4
Basp1
Sema5a
Slc12a2
Foxj1
Reck
Adamts12
Hoxa3
Adamts9
Sulf1
Rapgef2
Gaa
Obsl1
Prox1
Nrp2
Ttn
Fbn1
Eln
Folr1
Synpo2l
Kdm5b
Lrg1
Pml
Serpine2
Col15a1
Smo
Cav1
Fat4
Cdh13
Col4a2
c
Upregulation
ATP metabolic process (35)
Cell cycle (87)
Macromolecule catabolic process (71)
Transmembrane transport (75)
Cell activation (67)
Proteolysis (72)
Response to endogenous stimulus (65)
Cell activation involved in immune response (39)
Apoptotic process (68)
Myeloid leukocyte mediated immunity (30)
0 5 10 15 20
Enrichment score [-log10(P-value)]
e
Senp1-/- vs Senp1+/+
upregulated genes
670 278 148
Senp1-/-;Ripk1D138N/D138N vs Senp1-/-
downregulated genes
d
Downregulation
Circulatory system development (74)
Blood vessel morphogenesis (52)
Vasculature development (56)
Tube development (67)
Positive regulation of development (67)
Response to growth factor (47)
Cell-cell signaling by Wnt (36)
Actin filament based process (46)
Regulation of cell adhesion (42)
Epithelium development (57)
0 5 10 15 20 25
Enrichment score [-log10(P-value)]
h
Downregulated in Senp1-/- (vs Senp1+/+)
& not rescued in Senp1-/-;Ripk1D138N/D138N
Circulatory system development (45)
Tube development (41)
Vasculature development (33)
Blood vessel morphogenesis (31)
0 20 40 60 80
Enrichment score [-log10(P-value)]
f
Senp1+/-;Ripk1D138N/D138N × Senp1+/-;Ripk1D138N/D138N
Animals obtained | Mendelian expected | Observed
Senp1+/+;Ripk1D138N/D138N | 13 | 13
Senp1+/-;Ripk1D138N/D138N | 25 | 38
Senp1-/-;Ripk1D138N/D138N | 13 | 0
Total | 51 | 51
i
Senp1+/+
Senp1-/-
Senp1-/-;Ripk1D138N/D138N
EPO IHC
40 μm
Senp1+/+
Senp1-/-
Senp1-/-;Ripk1D138N/D138N
EPO expression (%area)
0 20 40 60
***P=1×10-14
P=0.999
j
Senp1+/+
Senp1-/-
Senp1-/-;Ripk1D138N/D138N
Relative mRNA levels
0.0 0.5 1.0 1.5
**P=3×10-3
***P=3×10-4
Epo
***P=5×10-4
***P=2×10-4
Epor

**Supplementary Figure 5. SENP1 suppresses RIPK1 activation during development**.

**a**, RIPK1 is hyperactivated in *Senp1*$^{-/-}$ fetal liver. Sections from E14.5 embryos of indicated genotype were immunostained for p-S166 RIPK1 and DAPI for nuclei. Representative images out of *n* = 8 embryos (for each genotype) are represented. **b**-**d**, Heatmap (**b**) and GO analysis (**c**, **d**) of genes differentially expressed (fold change > 1.5, *P*-value < 0.05) in E14.5 fetal livers of mice of indicated genotypes. *n* = 3 embryos for each genotype. **e**, Venn diagram of genes upregulated in *Senp1*$^{-/-}$ fetal livers (vs *Senp1*$^{+/+}$, fold change > 1.5, *P*-value < 0.05) and genes downregulated in *Senp1*$^{-/-}$*;Ripk1*$^{D138N/D138N}$ fetal livers (vs *Senp1*$^{-/-}$, fold change > 1.5, *P*-value < 0.05). **f**, Numbers of offspring from intercrossing *Senp1*$^{+/-}$;*Ripk1*$^{D138N/D138N}$ parents. **g**, **h**, RIPK1-D138N mutation does not alter the downregulation of genes that are involved in blood vessel development in *Senp1*$^{-/-}$ embryos. Heatmap (**g**) and GO analysis (**h**) of genes that are involved in blood vessel development, which are not rescued by RIPK1-D138N knockin mutation in *Senp1*$^{-/-}$ fetal livers. **i**, Immunohistochemistry and quantification of EPO in E14.5 fetal liver of indicated genotypes. Representative images out of *n* = 5 embryos (for each genotype) are represented. Graph depicting percentage of EPO expression area on fetal liver sections of indicated genotypes. Each dot represents an individual mouse. Mean ± s.e.m. One-way ANOVA, post hoc Dunnett's test. **j**, Quantitative RT-PCR analysis of the mRNA expression of *Epo* and *Epor* (*n* = 3 embryos for each genotype). Each dot represents an individual mouse. Mean ± s.e.m. One-way ANOVA, post hoc Dunnett's test. Source data are provided in Source Data file.

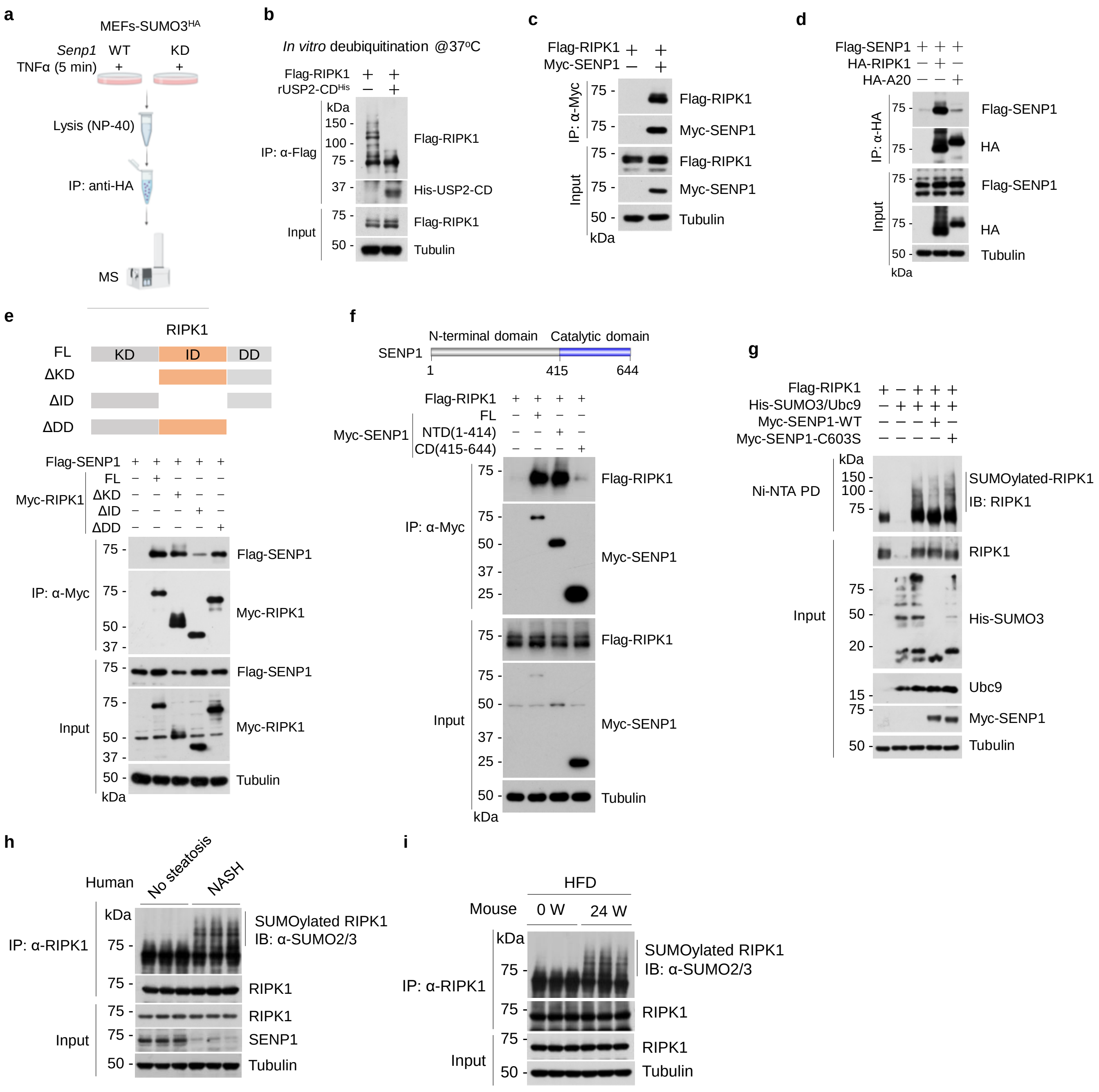
a
MEFs-SUMO3HA
Senp1 WT KD
TNFα (5 min) + +
Lysis (NP-40)
IP: anti-HA
MS
b
In vitro deubiquitination @37ºC
Flag-RIPK1 + +
rUSP2-CDHis − +
kDa
150
100
75
37
IP: α-Flag
Flag-RIPK1
His-USP2-CD
Input
Flag-RIPK1
Tubulin
c
Flag-RIPK1 + +
Myc-SENP1 − +
IP: α-Myc
Flag-RIPK1
Myc-SENP1
Input
Tubulin
kDa
d
Flag-SENP1 + + +
HA-RIPK1 − + −
HA-A20 − − +
IP: α-HA
Flag-SENP1
HA
Input
Tubulin
kDa
e
RIPK1
FL KD ID DD
ΔKD
ΔID
ΔDD
Flag-SENP1
Myc-RIPK1
IP: α-Myc
Flag-SENP1
Myc-RIPK1
Input
Tubulin
kDa
f
N-terminal domain Catalytic domain
SENP1
1 415 644
Flag-RIPK1
Myc-SENP1
FL
NTD(1-414)
CD(415-644)
IP: α-Myc
Flag-RIPK1
Myc-SENP1
Input
Tubulin
kDa
g
Flag-RIPK1
His-SUMO3/Ubc9
Myc-SENP1-WT
Myc-SENP1-C603S
Ni-NTA PD
SUMOylated-RIPK1
IB: RIPK1
Input
RIPK1
His-SUMO3
Ubc9
Myc-SENP1
Tubulin
h
Human
No steatosis
NASH
IP: α-RIPK1
SUMOylated RIPK1
IB: α-SUMO2/3
RIPK1
Input
RIPK1
SENP1
Tubulin
i
HFD
Mouse 0 W 24 W
IP: α-RIPK1
SUMOylated RIPK1
IB: α-SUMO2/3
RIPK1
Input
RIPK1
Tubulin

**Supplementary Figure 6. SENP1 interacts with RIPK1 and suppresses its SUMOylation**.

**a**, Schematic of mass spectrometry analysis for identification of SUMOylation targets in response to TNFα stimulation. Created with BioRender.com. **b**, HEK293T cells were transfected with Flag-RIPK1, and then immunoprecipitated using anti-Flag resin followed by incubation with recombinant catalytic domain (CD) of USP2 as indicated at 37 $^{o}$C for 1 hr. The samples were analyzed by immunoblotting with RIPK1. **c**, **d**, HEK293T cells were co-transfected with expression vectors of Flag-SENP1 and Myc-RIPK1 (**c**), Flag-SENP1 and HA-RIPK1 (**d**) or Flag-SENP1 and HA-A20 (**d**) as indicated for 24 hrs. The interaction between SENP1 and RIPK1 or SENP1 and A20 was revealed by immunoprecipitation-immunoblotting assay as indicated. **e**, HEK293T cells were co-transfected with expression vectors of Flag-SENP1 and Myc-tagged full-length RIPK1, or truncation mutants of RIPK1 as indicated for 24 hrs. The cell lysates were immunoprecipitated with anti-Flag conjugated beads. The interaction of SENP1 and RIPK1 truncation mutants was revealed by immunoblotting. KD: Kinase domain; ID: Intermediate domain; DD: Death domain. **f**, HEK293T cells were co-transfected with expression vectors of Flag-RIPK1 and Myc-tagged full-length SENP1 or truncation mutants of SENP1 as indicated for 24 hrs. The cell lysates were immunoprecipitated with anti-Myc conjugated beads. The interaction of RIPK1 and SENP1 truncation mutants was revealed by immunoblotting. NTD: N-terminal domain; CD: catalytic domain. **g**, HEK293T cells were co-transfected with expression plasmids of Flag-RIPK1, SUMOylaiton system (His-SUMO3 + Ubc9) and wild-type SENP1 (SENP1-WT) or its catalytic inactive mutant (SENP1-C603S) as indicated for 24 hr. The whole-cell lysate was subjected to pull-down with Ni-NTA agaroses, and SUMOylated RIPK1 was revealed by immunoblotting with anti-RIPK1 antibody. **h**, Representative western blot analysis of RIPK1 SUMOylation in the livers of individuals with non-steatosis ($n$ = 3) or NASH ($n$ = 3). **i**, Representative western blot analysis of RIPK1 SUMOylation in the livers of mice fed a HFD for the indicated number of weeks (W). $n$ = 3 mice per group. Similar results were obtained from $n$ = 3 independent experiments (**b-g**). Source data are provided in Source Data file.

**a**

| | |
|---|---|
| ...RLIFAGKQLEDGRTLSDYNIQKESTLHLVLRLRGG$_{76}$ | Ubiquitin |
| ...RFLFEGQRIADNHTPKELGMEEEDVIEVYQEQTGG$_{97}$ | SUMO1 |
| ...RFRFDGQPINETDTPAQLEMEDEDTIDVFQQQTGG$_{93}$ | SUMO2 |
| ...RFRFDGQPINETDTPAQLEMEDEDTIDVFQQQTGG$_{92}$ | SUMO3 |
| ...RFRFDGQPINETDTPAQLEMEDEDTIDVFQQQRGG$_{92}$ | SUMO3(T90R) |

**b**

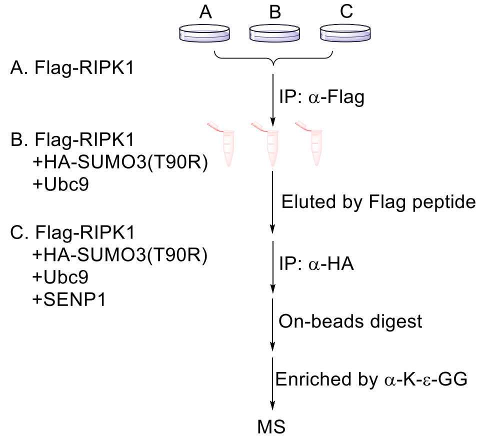


**c**

| Lysine (K) sites | Intensity | | | Ratio (C/B) |
|---|---|---|---|---|
| | A | B | C | |
| 550 | 0 | 331650000 | 78313000 | 0.24 |
| 132 | 0 | 212820000 | 20072000 | 0.09 |
| 185 | 0 | 66148000 | 10164000 | 0.15 |
| 226 | 0 | 38340000 | 15052000 | 0.39 |
| 167 | 0 | 16917000 | 1791100 | 0.11 |
| 205 | 0 | 12923000 | 4622600 | 0.36 |
| 11 | 0 | 4393700 | 0 | 0 |
| 619 | 0 | 3767500 | 1034300 | 0.27 |

**d**

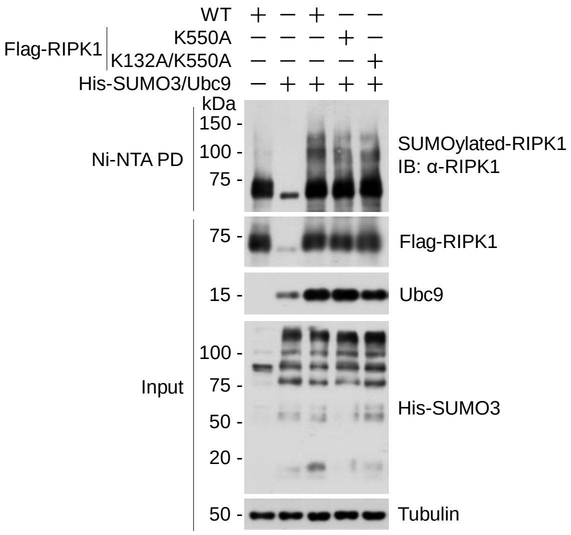


**e**

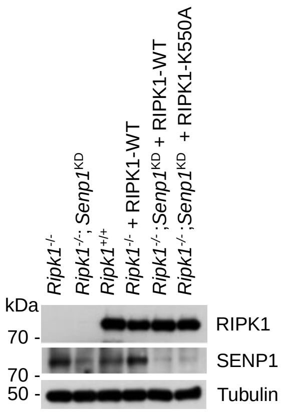


**f**

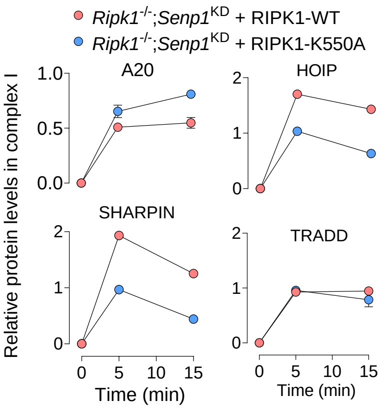

**Supplementary Figure 7. Lys550 is the main SUMOylation site of RIPK1**.

**a**, Sequence alignment of the C-terminal amino acid sequences of ubiquitin, SUMO1, SUMO2, SUMO3 and SUMO3-T90R mutant with the artificial introduction of a tryptic cleavage site. **b**, Schematic of mass spectrometry analysis for identification of SUMOylation site(s) of RIPK1. Details are described in Methods. **c**, RIPK1 SUMOylation sites identified by mass spectrometry. **d**, K550A mutation reduces the SUMOylation levels of RIPK1. HEK293T cells were co-transfected with the SUMOylation system (His-SUMO3 and Ubc9) and wild-type RIPK1 (RIPK1-WT) or its mutants as indicated for 24 hrs. The whole-cell lysate was subjected to pull-down with Ni-NTA agaroses, SUMOylated RIPK1 was revealed by immunoblotting with anti-RIPK1 antibody. Similar results were obtained from *n* = 3 independent experiments. **e**, Immunoblot analysis of RIPK1 and SENP1 expression in reconstituted lines. Similar results were obtained from *n* = 3 independent experiments. **f**, Quantification of indicated protein in TNF-RSC from Fig. 6d, data are represented as mean ± s.e.m of *n* = 3 independent experiments. Source data are provided in Source Data file.

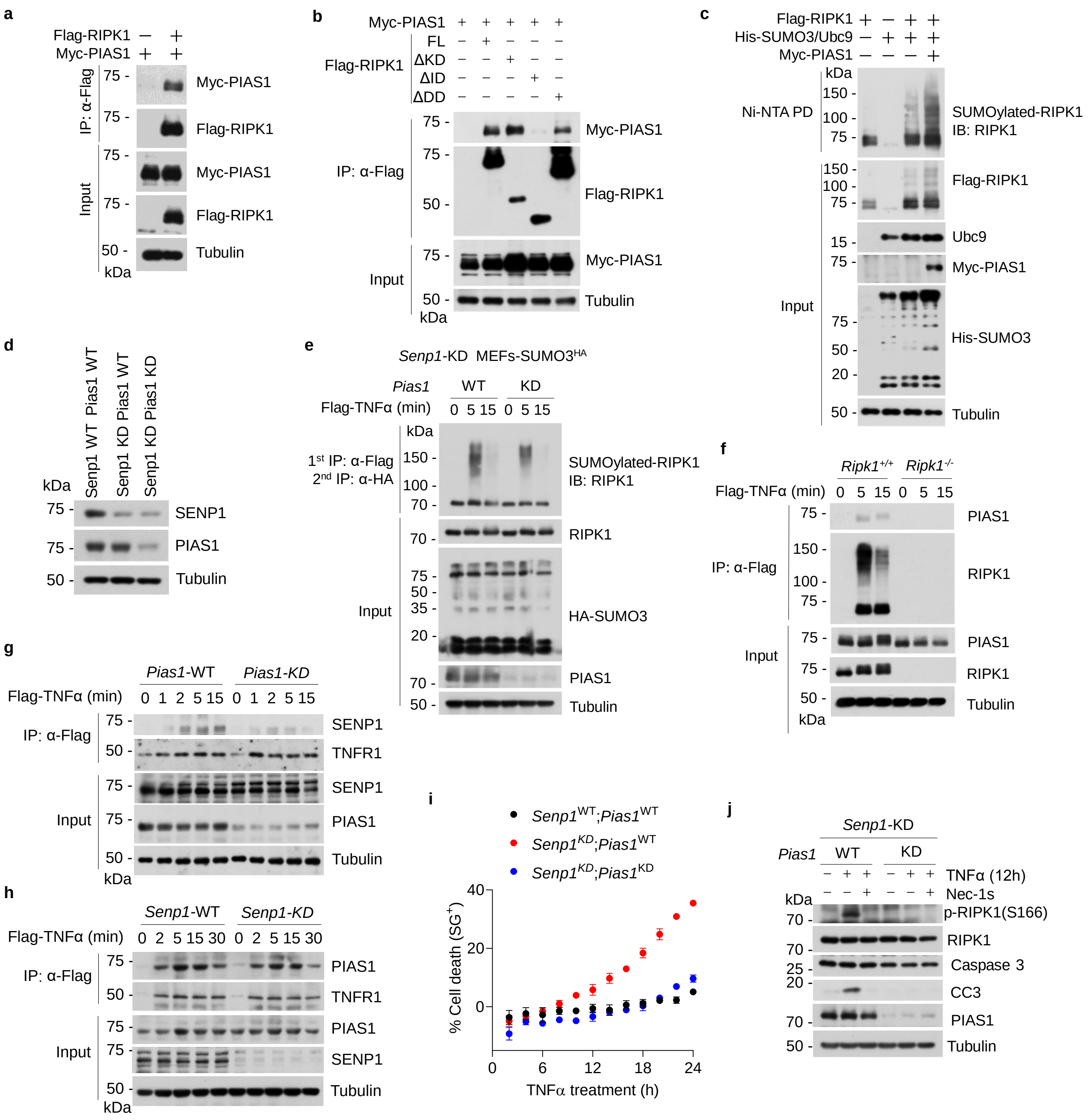
a
Flag-RIPK1 − +
Myc-PIAS1 + +
IP: α-Flag
Input
Myc-PIAS1
Flag-RIPK1
Myc-PIAS1
Flag-RIPK1
Tubulin
kDa
b
Myc-PIAS1 + + + + +
Flag-RIPK1
FL − + − − −
ΔKD − − + − −
ΔID − − − + −
ΔDD − − − − +
IP: α-Flag
Input
Myc-PIAS1
Flag-RIPK1
Myc-PIAS1
Tubulin
kDa
c
Flag-RIPK1 + − + +
His-SUMO3/Ubc9 − + + +
Myc-PIAS1 − − − +
Ni-NTA PD
SUMOylated-RIPK1
IB: RIPK1
Input
Flag-RIPK1
Ubc9
Myc-PIAS1
His-SUMO3
Tubulin
kDa
d
Senp1 WT Pias1 WT
Senp1 KD Pias1 WT
Senp1 KD Pias1 KD
kDa
SENP1
PIAS1
Tubulin
e
Senp1-KD MEFs-SUMO3HA
Pias1 WT KD
Flag-TNFα (min) 0 5 15 0 5 15
kDa
1st IP: α-Flag
2nd IP: α-HA
SUMOylated-RIPK1
IB: RIPK1
Input
RIPK1
HA-SUMO3
PIAS1
Tubulin
f
Ripk1+/+ Ripk1-/-
Flag-TNFα (min) 0 5 15 0 5 15
IP: α-Flag
Input
PIAS1
RIPK1
PIAS1
RIPK1
Tubulin
kDa
g
Pias1-WT Pias1-KD
Flag-TNFα (min) 0 1 2 5 15 0 1 2 5 15
IP: α-Flag
Input
SENP1
TNFR1
SENP1
PIAS1
Tubulin
kDa
h
Senp1-WT Senp1-KD
Flag-TNFα (min) 0 2 5 15 30 0 2 5 15 30
IP: α-Flag
Input
PIAS1
TNFR1
PIAS1
SENP1
Tubulin
kDa
i
Senp1WT;Pias1WT
Senp1KD;Pias1WT
Senp1KD;Pias1KD
% Cell death (SG+)
TNFα treatment (h)
0 6 12 18 24
0 20 40
j
Senp1-KD
Pias1 WT KD
− + + − + + TNFα (12h)
− − + − − + Nec-1s
kDa
p-RIPK1(S166)
RIPK1
Caspase 3
CC3
PIAS1
Tubulin

**Supplementary Figure 8. PIAS1 promotes RIPK1 SUMOylation in SENP1-deficient cells**.

**a**, HEK293T cells were co-transfected with expression vectors of Flag-RIPK1 and Myc-PIAS1 as indicated. The interaction between PIAS1 and RIPK1 was revealed by immunoprecipitation-immunoblotting assay. **b**, HEK293T cells were co-transfected with expression vectors of Myc-PIAS1 and Flag-tagged full-length RIPK1, or truncation mutants of RIPK1 as indicated for 24 hrs. The cell lysates were immunoprecipitated with anti-Flag conjugated beads. The interaction of PIAS1 and RIPK1 truncation mutants was revealed by immunoblotting. **c**, HEK293T cells were co-transfected with the indicated plasmids for 24 hrs. The whole-cell lysate was subjected to pull-down with Ni-NTA agaroses, RIPK1 SUMOylation was revealed by immunoblotting with anti-RIPK1 antibody. **d**, Immunoblot analysis of SENP1 and PIAS1 expression in SENP1 and PIAS1 double knockout cells. **e**, MEFs were stimulated by Flag-TNFα (100 ng/ml) for indicated time. RIPK1 SUMOylation was revealed by tandem immunoprecipitation of TNF-RSC and immunoblotting with anti-RIPK1 antibody. **f**, MEFs were stimulated with Flag-TNFα (100 ng/ml) for the indicated time. The recruitment of PIAS1 into TNF-RSC was analyzed by immunoblotting. **g**, **h**, PIAS1-WT and PIAS1-KD (**g**) or SENP1-WT and SENP1-KD (**h**) MEFs were stimulated with Flag-TNFα (100 ng/ml) for the indicated times. The recruitment of PIAS1 and SENP1 in TNF-RSC was immunoprecipitated via α-Flag beads and analysed by western blots. **i**, **j**, MEFs were treated with 10 ng/mL TNFα in the presence or absence of Nec-1s (10 μM) for 24 hrs. Cell death was measured as a function of time by SytoxGreen positivity assay. Data are presented as mean ± s.d. of three independent samples of one representative experiment out of $n$ = 3 independent experiments (**i**). The levels of p-S166 RIPK1 and CC3 were determined by immunoblotting (**j**). Similar results were obtained from $n$ = 3 independent experiments (**a**-**h**, **j**). Source data are provided in Source Data file.

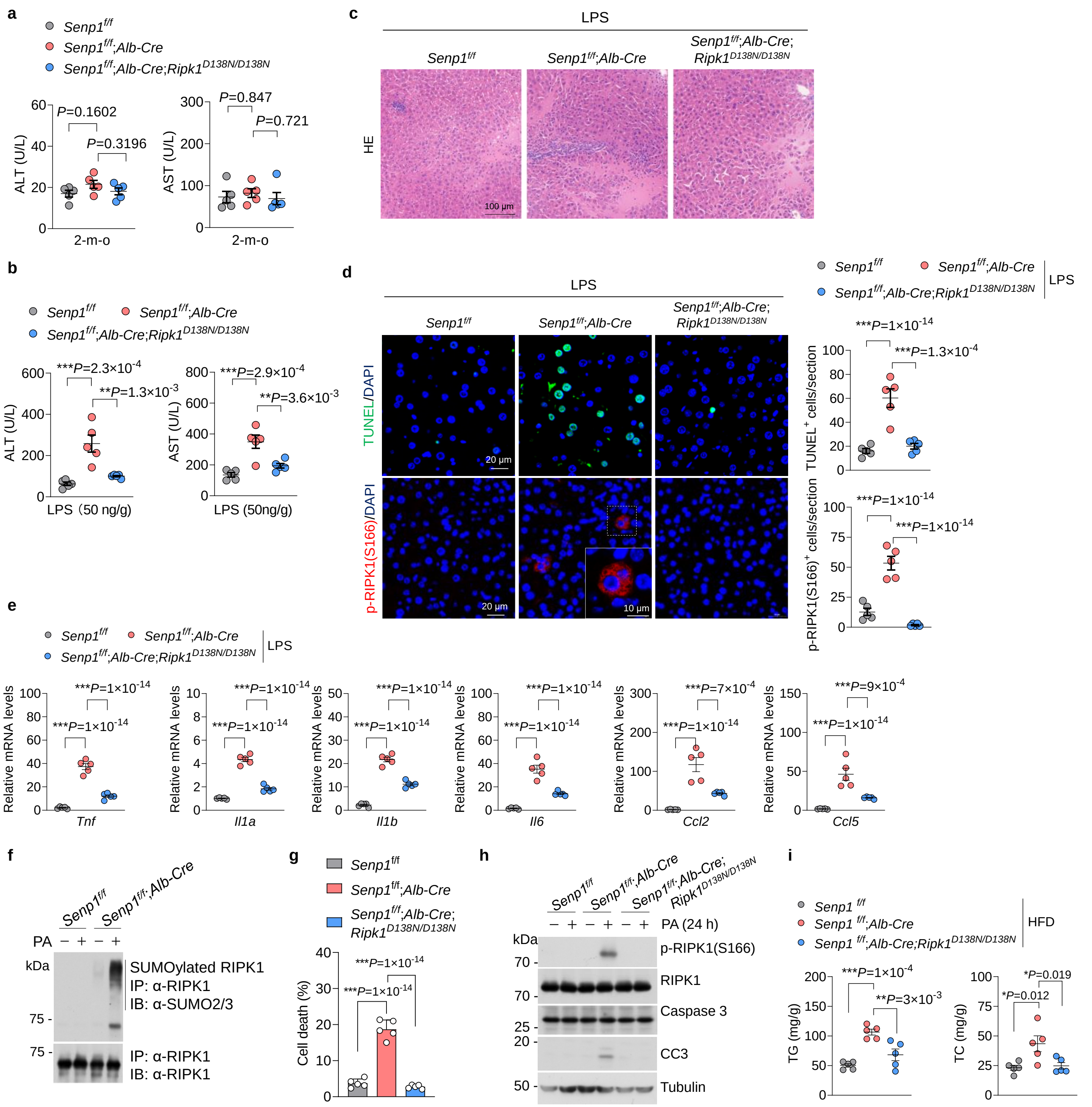

a
Senp1f/f
Senp1f/f;Alb-Cre
Senp1f/f;Alb-Cre;Ripk1D138N/D138N
ALT (U/L)
AST (U/L)
P=0.1602
P=0.3196
P=0.847
P=0.721
2-m-o
b
LPS (50 ng/g)
LPS (50ng/g)
***P=2.3×10-4
**P=1.3×10-3
***P=2.9×10-4
**P=3.6×10-3
c
LPS
HE
100 μm
d
TUNEL/DAPI
p-RIPK1(S166)/DAPI
20 μm
10 μm
TUNEL+ cells/section
p-RIPK1(S166)+ cells/section
***P=1×10-14
***P=1.3×10-4
e
Relative mRNA levels
Tnf
Il1a
Il1b
Il6
Ccl2
Ccl5
***P=7×10-4
***P=9×10-4
f
PA
kDa
SUMOylated RIPK1
IP: α-RIPK1
IB: α-SUMO2/3
IB: α-RIPK1
g
Cell death (%)
h
PA (24 h)
p-RIPK1(S166)
RIPK1
Caspase 3
CC3
Tubulin
i
HFD
TG (mg/g)
TC (mg/g)
***P=1×10-4
**P=3×10-3
*P=0.012
*P=0.019

**Supplementary Figure 9. SENP1 deficiency exaggerates LPS-induced acute liver injury in a RIPK1-dependent manner**.

**a**, Serum levels of ALT and AST of 2-m-o mice with indicated genotypes (*n* = 5 mice for each genotype). **b**, Plasma samples were harvested at 4 hrs after LPS (50 ng/g) injection for the measurement ALT and AST in 2-m-o mice with indicated genotypes (*n* = 5 for each genotype). **c**, HE staining of liver sections of mice from **b**. Representative images out of *n* = 5 mice for each genotype are represented. **d**, TUNEL assay and p-S166 RIPK1 staining of liver sections from mice treated with LPS (*n* = 5 for each genotype). Graph depicting numbers of $TUNEL^+$ cells and percentage of p-S166 $RIPK1^+$ staining on liver sections of indicated genotypes, respectively. **e**, Quantitative RT-PCR analysis of the mRNA expression of cytokines and chemokines in livers of mice from b (*n* = 5 mice for each genotype). **f**, Representative western blot analysis of RIPK1 SUMOylation levels in primary hepatocytes derived from 2-m-o mice with indicated genotypes treated with 0.4 mM PA for 24 hrs. Similar results were obtained from *n* = 3 independent experiments. **g**, **h**, SENP1 deficiency sensitized primary hepatocytes to cell death induced by PA. Primary hepatocytes derived from 2-m-o mice with indicated genotypes were treated with 0.4 mM PA for 24 hrs. Cell death was measured by SytoxGreen positivity assay, data are represented as mean ± s.d. of five independent samples of one representative experiment out of *n* = 3 independent experiments (**g**). The levels of p-S166 RIPK1 and CC3 were determined by immunoblotting, similar results were obtained from *n* = 3 independent experiments (**h**). **i**, Liver lipid content, including TG, TC content per gram of liver from 6-m-o mice with indicated genotypes fed with or without HFD for 4 months (*n* = 5 mice for each genotype). Data are presented as mean ± s.e.m. (**a**, **b**, **d**, **e**, **i**). One-way ANOVA, post hoc Dunnett's test (**a**, **b**, **d**, **e**, **g**, **i**). Source data are provided in Source Data file.
