# Supplementary material for: SENP1 prevents steatohepatitis by suppressing RIPK1-driven apoptosis and inflammation
Source: Nat Commun. 2022 Nov 22;13:7153. doi: 10.1038/s41467-022-34993-0 (PMC9681887; doi:10.1038/s41467-022-34993-0)
Supplement: Supplementary file 3 — Reporting summary [file 41467_2022_34993_MOESM3_ESM.pdf]

Corresponding author(s): Daichao Xu

Last updated by author(s): Oct 24, 2022

# Reporting Summary

Nature Portfolio wishes to improve the reproducibility of the work that we publish. This form provides structure for consistency and transparency in reporting. For further information on Nature Portfolio policies, see our Editorial Policies and the Editorial Policy Checklist.

## Statistics

For all statistical analyses, confirm that the following items are present in the figure legend, table legend, main text, or Methods section.

| n/a | Confirmed | |
|---|---|---|
| ☐ | ☒ | The exact sample size (*n*) for each experimental group/condition, given as a discrete number and unit of measurement |
| ☐ | ☒ | A statement on whether measurements were taken from distinct samples or whether the same sample was measured repeatedly |
| ☐ | ☒ | The statistical test(s) used AND whether they are one- or two-sided<br>*Only common tests should be described solely by name; describe more complex techniques in the Methods section.* |
| ☒ | ☐ | A description of all covariates tested |
| ☐ | ☒ | A description of any assumptions or corrections, such as tests of normality and adjustment for multiple comparisons |
| ☐ | ☒ | A full description of the statistical parameters including central tendency (e.g. means) or other basic estimates (e.g. regression coefficient) AND variation (e.g. standard deviation) or associated estimates of uncertainty (e.g. confidence intervals) |
| ☐ | ☒ | For null hypothesis testing, the test statistic (e.g. *F*, *t*, *r*) with confidence intervals, effect sizes, degrees of freedom and *P* value noted<br>*Give P values as exact values whenever suitable.* |
| ☒ | ☐ | For Bayesian analysis, information on the choice of priors and Markov chain Monte Carlo settings |
| ☒ | ☐ | For hierarchical and complex designs, identification of the appropriate level for tests and full reporting of outcomes |
| ☐ | ☒ | Estimates of effect sizes (e.g. Cohen's *d*, Pearson's *r*), indicating how they were calculated |

*Our web collection on statistics for biologists contains articles on many of the points above.*

## Software and code

Policy information about availability of computer code

| | |
|---|---|
| Data collection | Cell death assay: SYNERGY H1 microplate reader Gen5 software version 3.08.01 (BioTek)<br>q-RT-PCR:  QuantStudio 12K Flex software version 1.3 (Applied Biosystems)<br>Images: Leica Application Suite X software version 1.8.1.13759 (Leica)<br>RNA-seq data: llumina X-ten platforms software version HCS 3.3.76 (Illumina)<br>Gel blots scan: EPSON scan version 3.9.3.3SC (EPSON) |
| Data analysis | Results were analyzed using Prism version 8.4.1 (GraphPad Software).<br>Information from RNA-seq was analyzed by DESeq2 (version 1.26.0) program.<br>Gene term analyses were performed utilizing the online GSEA (4.1.0) program.<br>Images and quantifications of immunoblots were analyzed by ImageJ version 1.52a.<br>Mass spectrometry data analysis were performed by MaxQuant 1.2.1. |

For manuscripts utilizing custom algorithms or software that are central to the research but not yet described in published literature, software must be made available to editors and reviewers. We strongly encourage code deposition in a community repository (e.g. GitHub). See the Nature Portfolio guidelines for submitting code & software for further information.

# Data

Policy information about availability of data

All manuscripts must include a data availability statement. This statement should provide the following information, where applicable:

- Accession codes, unique identifiers, or web links for publicly available datasets
- A description of any restrictions on data availability
- For clinical datasets or third party data, please ensure that the statement adheres to our policy

The accession number for the sequencing data reported in this paper is GSE193509 (https://www.ncbi.nlm.nih.gov/geo/query/acc.cgi?acc=GSE193509). Sequencing reads were mapped to the reference genome mm10 (GRCm38: GCA_000001635.8, GCF_000001635.26) from Gencode with hisat2 by default parameter. The accession code for mass spectrometry proteomics data is PXD037648 (http://proteomecentral.proteomexchange.org/cgi/GetDataset?ID=PXD037648).These data are available with no restriction. The clinical and histological characteristics of the human samples are not publicly available due to data privacy, but that anonymized data can be obtained from the corresponding author. Source data are provided with this paper.

# Human research participants

Policy information about studies involving human research participants and Sex and Gender in Research.

| | |
|---|---|
| Reporting on sex and gender | No sex and gender analyses were performed. |
| Population characteristics | 31 male patients and 4 female patients with different stages of NAFLD were enrolled for this study. All the patients are Asian. The clinical and histological characteristics of the human samples are not publicly available due to data privacy, but that anonymized data can be obtained from the corresponding author. |
| Recruitment | Steatotic livers were obtained from individuals with NAFLD or NASH who underwent liver biopsy or steatotic liver grafts from China Donation after Citizen's Death. Liver steatosis due to excessive alcohol consumption (>140 g for men or >70 g for women, per week), use of toxins or drugs, and viral infection (for example, hepatitis B virus and hepatitis C virus) were excluded from the study. Samples from nonsteatotic liver were collected from the normal donor livers. All donor livers were allocated via China Organ Transplant Response System from 2017 to 2021. The donors were enrolled in the study on a volunteer basis, and the families of organ donors were approached for consent. Written informed consent was obtained from subjects or families of all participants. |
| Ethics oversight | All procedures involving human samples were approved by the Ethics Committee of Xinhua Hospital affiliated to Shanghai Jiao Tong University School of Medicine (Approval no. XHEC-D-2022-040), and were consistent with the principles outlined in the Declaration of Helsinki. |

Note that full information on the approval of the study protocol must also be provided in the manuscript.

# Field-specific reporting

Please select the one below that is the best fit for your research. If you are not sure, read the appropriate sections before making your selection.

☒ Life sciences ☐ Behavioural & social sciences ☐ Ecological, evolutionary & environmental sciences

For a reference copy of the document with all sections, see nature.com/documents/nr-reporting-summary-flat.pdf

# Life sciences study design

All studies must disclose on these points even when the disclosure is negative.

| | |
|---|---|
| Sample size | For cell culture-based experiments, sample sizes were determined based on pilot experiments and previous experience with the models and the methods used in this study: PMID: 30146158. The sample sizes for animal-based experiments were chosen based on generally expected variations of metabolic parameters and typical sample sizes for metabolic studies documented in literature (PMID: 29227477). Sample numbers were well described in the Figure legends. |
| Data exclusions | No data were excluded from the analyses. |
| Replication | All the biological experiments were repeated, at least, three times and reproduced. Western blotting data were confirmed by at least three independent samples. |
| Randomization | For animal experiments, age-matched mice with different genotypes were randomly divided into different experimental groups. For cell culture experiments, cells with different genotypes or treatments were randomly divided into different experimental groups. |
| Blinding | For in vitro experiments, blinding was difficult to apply, since experiments were performed by the same investigator. However, all experiments were independently performed mutiple times to ensure careful interpretation of the results. For animal experiments, the operators were not blinded to the genotyping information, because the operator mainly raised and genotyped these mice. However, the |

investigators were blinded to the genotyping information during data collection and analysis, included immunofluorescence experiments, histological staining experiments.

# Reporting for specific materials, systems and methods

We require information from authors about some types of materials, experimental systems and methods used in many studies. Here, indicate whether each material, system or method listed is relevant to your study. If you are not sure if a list item applies to your research, read the appropriate section before selecting a response.

## Materials & experimental systems

| n/a | Involved in the study |
|---|---|
| ☐ | ☒ Antibodies |
| ☐ | ☒ Eukaryotic cell lines |
| ☒ | ☐ Palaeontology and archaeology |
| ☐ | ☒ Animals and other organisms |
| ☒ | ☐ Clinical data |
| ☒ | ☐ Dual use research of concern |

## Methods

| n/a | Involved in the study |
|---|---|
| ☒ | ☐ ChIP-seq |
| ☒ | ☐ Flow cytometry |
| ☒ | ☐ MRI-based neuroimaging |

## Antibodies

Antibodies used

p-S166 RIPK1 Biolynx Cat#BX60008 YJY-1-5
RIPK1 Cell Signaling Technology Cat#3493 D94C12
PIAS1 Cell Signaling Technology Cat#3550 D33A7
A20/TNFAIP3 Cell Signaling Technology Cat#5630 D13H3
TNFR1 Cell Signaling Technology Cat#13377 D317K
Cleaved Caspase-3 Cell Signaling Technology Cat#9661
p-p38 MAPK Cell Signaling Technology Cat#9216 28B10
p-SAPK/JNK Cell Signaling Technology Cat#4671 98F2
p-IκBα Cell Signaling Technology Cat#2859 14D4
IκBα Cell Signaling Technology Cat#4814 L35A5
HA-tag Cell Signaling Technology Cat#3724 C29F4
RIPK3 Cell Signaling Technology Cat#95702 D4G2A
p-Thr231/Ser232 RIPK3 Cell Signaling Technology Cat#91702 E7S1R
p-S345 MLKL Abcam Cat#ab196436 EPR9515(2)
RNF31/HOIP Abcam Cat#ab46322
FADD Abcam Cat#ab124812 EPR5030
FADD Santa Cruz Cat#sc-6036
SENP1 Santa Cruz Cat#sc-271360 C-12
SENP1 Abcam Cat#ab108981
ERK1/2 Proteintech Cat#67170-1-Ig 1A3D3
JNK Proteintech Cat#24164-1-AP
p38 Proteintech Cat#14064-1-AP
Caspase3 Proteintech Cat#19677-1-AP
SAE1 Proteintech Cat#10229-1-AP
SHARPIN Proteintech Cat#14626-1-AP
β-Tubulin TRANS Cat#HC101-02
SUMO1/2/3 PTM-BIO Cat#PTM-1109
Flag-tag Sigma Cat#F7425
Myc-tag Sigma Cat#C3956
CD45 Servicebio Cat#GB11066
EPO Servicebio Cat#GB11323
Alexa Fluor 488 goat anti-rabbit IgG  Invitrogen Cat#A11034
Alexa Fluor 568 goat anti-rabbit IgG Invitrogen Cat#A11011

Validation

p-S166 RIPK1 Biolynx Cat#BX60008 YJY-1-5
http://www.biolynxtec.com/products/antibody/p-ripk1s166.html
Species specificity: Mouse
Applications: WB, IP, IF/ICC, IHC-Fr
Publications: PMID: 30146158

RIPK1 Cell Signaling Technology Cat#3493 D94C12
https://www.cellsignal.cn/products/primary-antibodies/rip-d94c12-xp-rabbit-mab/3493?site-search-type=Products&N=4294956287&Ntt=3493&fromPage=plp&_requestid=1999609
Species specificity: Human, mouse, rat, hamster, monkey
Applications: WB, IP, IF, F
Publications: PMID: 35831301, 35658939, 35641486

PIAS1 Cell Signaling Technology Cat#3550 D33A7
https://www.cellsignal.cn/products/primary-antibodies/pias1-d33a7-xp-rabbit-mab/3550?site-search-

type=Products&N=4294956287&Ntt=3550&fromPage=plp&_requestid=2003556
Species specificity: Human, mouse, rat, monkey
Applications: WB, IF, F
Publications: PMID: 32827359, 32937131, 32047143

A20/TNFAIP3 Cell Signaling Technology Cat#5630 D13H3
https://www.cellsignal.cn/products/primary-antibodies/a20-tnfaip3-d13h3-rabbit-mab/5630?site-search-type=Products&N=4294956287&Ntt=5630&fromPage=plp&_requestid=2004705
Species specificity: Human, mouse, rat, monkey
Applications: WB, IP
Publications: PMID: 34853447, 35430614, 35120571

TNFR1 Cell Signaling Technology Cat#13377 D317K
https://www.cellsignal.cn/products/primary-antibodies/tnf-r1-d3i7k-rabbit-mab-rodent-specific/13377?site-search-type=Products&N=4294956287&Ntt=13377&fromPage=plp&_requestid=2005869
Species specificity: Mouse, rat
Applications: WB
Publications: PMID: 34376696, 33914027, 33152324

Cleaved Caspase-3 Cell Signaling Technology Cat#9661
https://www.cellsignal.cn/products/primary-antibodies/cleaved-caspase-3-asp175-antibody/9661?site-search-type=Products&N=4294956287&Ntt=9661&fromPage=plp&_requestid=2006946
Species specificity: Human, mouse, rat, monkey
Applications: WB, IP, IHC, IF, F
Publications: PMID: 35416106, 35894142, 35644004

p-p38 MAPK Cell Signaling Technology Cat#9216 28B10
https://www.cellsignal.cn/products/primary-antibodies/phospho-p38-mapk-thr180-tyr182-28b10-mouse-mab/9216?site-search-type=Products&N=4294956287&Ntt=9216&fromPage=plp&_requestid=2008392
Species specificity: Human, mouse, rat, monkey, Saccharomyces cerevisiae
Applications: WB, IP, IF, F
Publications: PMID: 35903325, 35897640, 35831876

p-SAPK/JNK Cell Signaling Technology Cat#4671 98F2
https://www.cellsignal.cn/products/primary-antibodies/phospho-sapk-jnk-thr183-tyr185-98f2-rabbit-mab/4671?site-search-type=Products&N=4294956287&Ntt=4671&fromPage=plp&_requestid=2010478
Species specificity: Human, mouse, rat, hamster
Applications: WB
Publications: PMID: 35842613, 35831279, 35785771

p-IκBα Cell Signaling Technology Cat#2859 14D4
https://www.cellsignal.cn/products/primary-antibodies/phospho-ikba-ser32-14d4-rabbit-mab/2859?_=1661225107379&Ntt=2859&tahead=true
Species specificity: Human, mouse, rat, monkey
Applications: WB, IP
Publications: PMID: 35873013, 35674157, 35702666

IκBα Cell Signaling Technology Cat#4814 L35A5
https://www.cellsignal.cn/products/primary-antibodies/ikba-l35a5-mouse-mab-amino-terminal-antigen/4814?site-search-type=Products&N=4294956287&Ntt=4814&fromPage=plp
Species specificity: Human, mouse, rat, monkey, pig
Applications: WB, IP, IHC, IF, F
Publications: PMID: 35873013, 35874657, 35871708

HA-tag Cell Signaling Technology Cat#3724 C29F4
https://www.cellsignal.cn/products/primary-antibodies/ha-tag-c29f4-rabbit-mab/3724?site-search-type=Products&N=4294956287&Ntt=3724&fromPage=plp&_requestid=2037503
Species specificity: All
Applications: WB, IP, IHC, IF, F, CHIP
Publications: PMID: 35732914, 35906225, 35881485

RIPK3 Cell Signaling Technology Cat#95702 D4G2A
https://www.cellsignal.cn/products/primary-antibodies/rip3-d4g2a-rabbit-mab/95702?site-search-type=Products&N=4294956287&Ntt=95702&fromPage=plp&_requestid=2038936
Species specificity: Mouse
Applications: WB, IP, IF, F
Publications: PMID: 35873796, 35533409, 35711363

p-Thr231/Ser232 RIPK3 Cell Signaling Technology Cat#91702 E7S1R
https://www.cellsignal.cn/products/primary-antibodies/phospho-rip3-thr231-ser232-e7s1r-rabbit-mab/91702?site-search-type=Products&N=4294956287&Ntt=91702&fromPage=plp&_requestid=2039746
Species specificity: Mouse
Applications: WB, IF
Publications: PMID: 35533409, 35648750, 35587515

p-S345 MLKL Abcam Cat#ab196436 EPR9515(2)

https://www.abcam.cn/mlkl-phospho-s345-antibody-epr95152-ab196436.html
Species specificity: Mouse
Applications: WB, IP, Dot blot
Publications: PMID: 32999468, 33386754, 33431801

RNF31/HOIP Abcam Cat#ab46322
https://www.abcam.cn/rnf31hoip-antibody-ab46322.html
Species specificity: Human
Applications: ICC/IF, WB
Publications: PMID: 33824292, 31534131, 30936877

FADD Abcam Cat#ab124812 EPR5030
https://www.abcam.cn/fadd-antibody-epr5030-ab124812.html
Species specificity: Mouse
Applications: Flow Cyt (Intra), WB, IHC-P, ICC, IP
Publications: PMID: 31949127, 32411316, 32428502

FADD Santa Cruz Cat#sc-6036
https://www.scbt.com/p/fadd-antibody-m-19?requestFrom=search
Species specificity: Mouse
Applications: IP
Publications: PMID: 24439895, 22362767, 21052097

SENP1 Santa Cruz Cat#sc-271360 C-12
https://www.scbt.com/p/senp1-antibody-c-12?requestFrom=search
Species specificity: Mouse, rat, human
Applications: WB, IP, IF, ELISA
Publications: PMID: 35734580, 35186948, 34462260

SENP1 Abcam Cat#ab108981
https://www.abcam.cn/senp1-antibody-epr3844-ab108981.html
Species specificity: Human
Applications: Flow Cyt (Intra), ICC/IF, WB, IHC-P
Publications: PMID: 33753739, 33795649, 33577677

ERK1/2 Proteintech Cat#67170-1-Ig 1A3D3
https://www.ptgcn.com/products/ERK1-2-Antibody-67170-1-Ig.htm
Species specificity: Human, mouse, rat
Applications: ELISA, IHC, WB
Publications: PMID: 35925441, 33571699, 34326318

JNK Proteintech Cat#24164-1-AP
https://www.ptgcn.com/products/JNK-Antibody-24164-1-AP.htm#product-information
Species specificity: Human, mouse
Applications: IF, IHC, WB, ELISA
Publications: PMID: 30598514, 30885574, 31033175

p38 Proteintech Cat#14064-1-AP
https://www.ptgcn.com/products/p38-Antibody-14064-1-AP.htm#product-information
Species specificity: Human, mouse, rat
Applications: IF, IHC, WB, ELISA
Publications: PMID: 30598514, 30670092, 30670969

Caspase3 Proteintech Cat#19677-1-AP
https://www.ptgcn.com/products/CASP3-Antibody-19677-1-AP.htm
Species specificity: Human, mouse, rat
Applications: FC, IF, IHC, IP, WB, ELISA
Publications: PMID: 30568466, 35873644, 34570444

SAE1 Proteintech Cat#10229-1-AP
https://www.ptgcn.com/products/SAE1-Antibody-10229-1-AP.htm
Species specificity: Human, mouse, rat
Applications: IF, IHC, WB, ELISA
Publications: PMID: 30652970, 35859792, 22342750

SHARPIN Proteintech Cat#14626-1-AP
https://www.ptgcn.com/products/SHARPIN-Antibody-14626-1-AP.htm
Species specificity: Human, mouse, rat
Applications: IF, IHC, IP, WB, ELISA
Publications: PMID: 30901564, 30988283, 30913450

β-Tubulin TRANS Cat#HC101-02
https://www.transgen.com.cn/antibody_reference/391.html
Species specificity: Human, mouse, rat
Applications: WB, ELISA, IF, IP
Publications: PMID: 28467790

SUMO1/2/3 PTM-BIO Cat#PTM-1109
http://www.ptm-biolab.com.cn/productDetail.html?id=5644
Species specificity: Mouse
Applications: WB, IHC, ICC/IF
Publications: PMID: 32980686

Flag-tag Sigma Cat#F7425
https://www.sigmaaldrich.cn/CN/zh/product/sigma/f7425
Species specificity: All
Applications: dot blot, IP, IF, WB
Publications: PMID: 31456775, 24045953, 31217244

Myc-tag Sigma Cat#C3956
https://www.sigmaaldrich.cn/CN/zh/product/sigma/c3956
Species specificity: Human
Applications: IP, IF, WB
Publications: PMID: 17034881, 22651703, 20826535

CD45 Servicebio Cat#GB11066
https://www.servicebio.cn/goodsdetail?id=1349
Species specificity: Mouse
Applications: IHC, IF
Publications: PMID: 32873321, 32512518, 29669342

EPO Servicebio Cat#GB11323
https://www.servicebio.cn/goodsdetail?id=1151
Species specificity: Mouse, Rat
Applications: WB, IHC, IF
Publications: validated by this paper

Goat anti-Rabbit IgG (H+L) Highly Cross-Adsorbed Secondary Antibody, Alexa Fluor™ 488 Cat#A11034
https://www.thermofisher.cn/cn/zh/antibody/product/Goat-anti-Rabbit-IgG-H-L-Highly-Cross-Adsorbed-Secondary-Antibody-Polyclonal/A-11034
Species specificity: Rabbit
Applications: ICC/IF, Flow
Publications: PMID: 36093836, 36250205, 36189424

Goat anti-Rabbit IgG (H+L) Cross-Adsorbed Secondary Antibody, Alexa Fluor™ 568
Cat# A-11011
https://www.thermofisher.cn/cn/zh/antibody/product/Goat-anti-Rabbit-IgG-H-L-Cross-Adsorbed-Secondary-Antibody-Polyclonal/A-11011
Species specificity: Rabbit
Applications: IHC (F), ICC/IF, Flow
Publications: PMID: 35662224, 36229429, 36229454

## Eukaryotic cell lines

Policy information about cell lines and Sex and Gender in Research

| | |
|---|---|
| Cell line source(s) | HEK293T ATCC CRL3216<br>MEFs ATCC CRL-2991 |
| Authentication | All cell lines were verified by ATCC. All cell lines were authenticated in our lab by morphological examination using microscope and were not authenticated again genetically. |
| Mycoplasma contamination | The cells were tested every two months by a TransDetect PCR Mycroplasma Detection Kit (Transgen Biotech, cat. no. FM311-01) to ensure that they are mycoplasma free. |
| Commonly misidentified lines<br>(See ICLAC register) | There is no ICLAC line used in this study. |

## Animals and other research organisms

Policy information about studies involving animals; ARRIVE guidelines recommended for reporting animal research, and Sex and Gender in Research

| | |
|---|---|
| Laboratory animals | ·Senp1+/- mice, Senp1-flox/flox mice were kindly provided by Professor Jinke Cheng (Shanghai Jiao Tong University College of Basic Medical Sciences, China).<br>·Alb-Cre mice were from The Jackson Laboratory (Catalog No. 003574).<br>·Ripk1-D138N/D138N mice were generated as previous reported (PMID: 32513687).<br>·Senp1+/- mice were crossed with Ripk1-D138N/D138N mice to generate Senp1+/-;Ripk1-D138N/D138N mice.<br>·Senp1-flox/flox;Alb-Cre mice were crossed with Ripk1-D138N/D138N mice to generate Senp1-flox/flox;Alb-Cre;Ripk1-D138N/D138N |

mice.
·All mice were in the C57BL/6J background.
·For mouse embryo-related experiments, E14.5 embryos (Senp1+/+, Senp1-/- and Senp1-/-;Ripk1-D138N/D138N) were used.
·For spontaneous NASH-related phenotype analysis, 3-month-old or 8-month-old mice (Senp1-flox/flox, Senp1-flox/flox;Alb-Cre, and Senp1-flox/flox;Alb-Cre;Ripk1-D138N/D138N) were used.
·For high-fat-diet (HFD)-induced NAFLD mouse model, 8-week-old male mice (Senp1-flox/flox, Senp1-flox/flox;Alb-Cre, and Senp1-flox/flox;Alb-Cre;Ripk1-D138N/D138N) were used.
·All animals were maintained in a specific pathogen-free environment, and were kept under controlled light (12 hour light and 12 hour dark cycle), temperature (24 ± 2oC) and humidity (50% ± 10%) conditions.

Wild animals

This study did not involve wild animals

Reporting on sex

To reduce the effect of sex variance, male mice aged 8 weeks were used for HFD-induced NAFLD experiments.

Field-collected samples

This study did not involve samples collected from the field.

Ethics oversight

All animals were maintained in a specific pathogen-free environment, and animal experiments were conducted according to the protocols approved by the Standing Animal Care Committee at the Interdisciplinary Research Center on Biology and Chemistry (Approval no. ECSIOC2021-08).

Note that full information on the approval of the study protocol must also be provided in the manuscript.
